# Supplementary material for: Resolving the full spectrum of human genome variation using Linked-Reads
Source: Genome Res. 2019 Apr;29(4):635–45. doi: 10.1101/gr.234443.118 (PMC6442396; doi:10.1101/gr.234443.118)
Supplement: Supplemental Material [file supp_gr.234443.118_Supplemental_longranger-2.2.zip › longranger-2.2.tar.bz2!/lr2.2-public/lib/stan/cmdstan/stan/doc/api/html/multi__student__t_8hpp_source.html]

Stan: src/stan/prob/distributions/multivariate/continuous/multi\_student\_t.hpp Source File


|  |  |
| --- | --- |
|  | Stan  2.5.0  probability, sampling & optimization |


- Main Page
- Related Pages
- Namespaces
- Classes
- Files

- File List
- File Members

All Classes Namespaces Files Functions Variables Typedefs Enumerations Enumerator Friends Macros Pages

- src
- stan
- prob
- distributions
- multivariate
- continuous

multi\_student\_t.hpp

Go to the documentation of this file.

1 #ifndef STAN\_\_PROB\_\_DISTRIBUTIONS\_\_MULTI\_STUDENT\_T\_HPP

2 #define STAN\_\_PROB\_\_DISTRIBUTIONS\_\_MULTI\_STUDENT\_T\_HPP

3

4 #include <cstdlib>

5

6 #include <boost/math/special\_functions/gamma.hpp>

7 #include <boost/math/special\_functions/fpclassify.hpp>

8

9 #include <stan/math/matrix.hpp>

10 #include <stan/math/error\_handling.hpp>

11 #include <stan/math/matrix/multiply.hpp>

12 #include <stan/math/matrix/dot\_product.hpp>

13 #include <stan/math/matrix/subtract.hpp>

14 #include <stan/math/matrix\_error\_handling.hpp>

15 #include <stan/prob/constants.hpp>

16 #include <stan/prob/traits.hpp>

17 #include <stan/prob/distributions/multivariate/continuous/multi\_normal.hpp>

18 #include <stan/prob/distributions/univariate/continuous/inv\_gamma.hpp>

19 #include <stan/math/error\_handling/matrix/check\_ldlt\_factor.hpp>

20 #include <boost/random/variate\_generator.hpp>

21

22 namespace stan {

23

24  namespace prob {

25

32  template <bool propto,

33  typename T\_y, typename T\_dof, typename T\_loc, typename T\_scale>

34  typename boost::math::tools::promote\_args<typename scalar\_type<T\_y>::type,T\_dof,typename scalar\_type<T\_loc>::type,T\_scale>::type

35  multi\_student\_t\_log(const T\_y& y,

36  const T\_dof& nu,

37  const T\_loc& mu,

38  const

39  Eigen::Matrix<T\_scale,

40  Eigen::Dynamic,Eigen::Dynamic>& Sigma) {

41  static const char\* function = "stan::prob::multi\_student\_t(%1%)";

42

43  using stan::math::check\_size\_match;

44  using stan::math::check\_finite;

45  using stan::math::check\_not\_nan;

46  using stan::math::check\_symmetric;

47  using stan::math::check\_positive;

48  using boost::math::tools::promote\_args;

49  using boost::math::lgamma;

50  using stan::math::log\_determinant\_ldlt;

51  using stan::math::LDLT\_factor;

52  using stan::math::check\_ldlt\_factor;

53

54  typedef typename boost::math::tools::promote\_args<typename scalar\_type<T\_y>::type,T\_dof,typename scalar\_type<T\_loc>::type,T\_scale>::type lp\_type;

55  lp\_type lp(0.0);

56

57  // allows infinities

58  check\_not\_nan(function, nu,

59  "Degrees of freedom parameter", &lp);

60  check\_positive(function, nu,

61  "Degrees of freedom parameter", &lp);

62

63  using boost::math::isinf;

64

65  if (isinf(nu)) // already checked nu > 0

66  return multi\_normal\_log(y,mu,Sigma);

67

68  using Eigen::Matrix;

69  using Eigen::Dynamic;

70  using std::vector;

71  VectorViewMvt<const T\_y> y\_vec(y);

72  VectorViewMvt<const T\_loc> mu\_vec(mu);

73  //size of std::vector of Eigen vectors

74  size\_t size\_vec = max\_size\_mvt(y, mu);

75

76

77  //Check if every vector of the array has the same size

78  int size\_y = y\_vec[0].size();

79  int size\_mu = mu\_vec[0].size();

80  if (size\_vec > 1) {

81  int size\_y\_old = size\_y;

82  int size\_y\_new;

83  for (size\_t i = 1, size\_ = length\_mvt(y); i < size\_; i++) {

84  int size\_y\_new = y\_vec[i].size();

85  check\_size\_match(function,

86  size\_y\_new, "Size of one of the vectors of the random variable",

87  size\_y\_old, "Size of another vector of the random variable",

88  &lp);

89  size\_y\_old = size\_y\_new;

90  }

91  int size\_mu\_old = size\_mu;

92  int size\_mu\_new;

93  for (size\_t i = 1, size\_ = length\_mvt(mu); i < size\_; i++) {

94  int size\_mu\_new = mu\_vec[i].size();

95  check\_size\_match(function,

96  size\_mu\_new, "Size of one of the vectors of the location variable",

97  size\_mu\_old, "Size of another vector of the location variable",

98  &lp);

99  size\_mu\_old = size\_mu\_new;

100  }

101  (void) size\_y\_old;

102  (void) size\_y\_new;

103  (void) size\_mu\_old;

104  (void) size\_mu\_new;

105  }

106

107

108  check\_size\_match(function,

109  size\_y, "Size of random variable",

110  size\_mu, "size of location parameter",

111  &lp);

112  check\_size\_match(function,

113  size\_y, "Size of random variable",

114  Sigma.rows(), "rows of scale parameter",

115  &lp);

116  check\_size\_match(function,

117  size\_y, "Size of random variable",

118  Sigma.cols(), "columns of scale parameter",

119  &lp);

120

121  for (size\_t i = 0; i < size\_vec; i++) {

122  check\_finite(function, mu\_vec[i], "Location parameter", &lp);

123  check\_not\_nan(function, y\_vec[i], "Random variable", &lp);

124  }

125  check\_symmetric(function, Sigma, "Scale parameter", &lp);

126

127

128  LDLT\_factor<T\_scale,Eigen::Dynamic,Eigen::Dynamic> ldlt\_Sigma(Sigma);

129  check\_ldlt\_factor(function,ldlt\_Sigma,"LDLT\_Factor of scale parameter",

130  &lp);

131

132  if (size\_y == 0) //y\_vec[0].size() == 0

133  return lp;

134

135  if (include\_summand<propto,T\_dof>::value) {

136  lp += lgamma(0.5 \* (nu + size\_y)) \* size\_vec;

137  lp -= lgamma(0.5 \* nu) \* size\_vec;

138  lp -= (0.5 \* size\_y) \* log(nu) \* size\_vec;

139  }

140

141  if (include\_summand<propto>::value)

142  lp -= (0.5 \* size\_y) \* LOG\_PI \* size\_vec;

143

144  using stan::math::multiply;

145  using stan::math::dot\_product;

146  using stan::math::subtract;

147  using Eigen::Array;

148

149

150  if (include\_summand<propto,T\_scale>::value) {

151  lp -= 0.5 \* log\_determinant\_ldlt(ldlt\_Sigma) \* size\_vec;

152  }

153

154  if (include\_summand<propto,T\_y,T\_dof,T\_loc,T\_scale>::value) {

155  lp\_type sum\_lp\_vec(0.0);

156  for (size\_t i = 0; i < size\_vec; i++) {

157  Matrix<typename

158  boost::math::tools::promote\_args<typename scalar\_type<T\_y>::type, typename scalar\_type<T\_loc>::type>::type,

159  Dynamic, 1> y\_minus\_mu(size\_y);

160  for (int j = 0; j < size\_y; j++)

161  y\_minus\_mu(j) = y\_vec[i](j)-mu\_vec[i](j);

162  sum\_lp\_vec += log(1.0 + trace\_inv\_quad\_form\_ldlt(ldlt\_Sigma,y\_minus\_mu) / nu);

163  }

164  lp -= 0.5 \* (nu + size\_y) \* sum\_lp\_vec;

165  }

166  return lp;

167  }

168

169  template <typename T\_y, typename T\_dof, typename T\_loc, typename T\_scale>

170  inline

171  typename boost::math::tools::promote\_args<typename scalar\_type<T\_y>::type,T\_dof,typename scalar\_type<T\_loc>::type,T\_scale>::type

172  multi\_student\_t\_log(const T\_y& y,

173  const T\_dof& nu,

174  const T\_loc& mu,

175  const

176  Eigen::Matrix<T\_scale,

177  Eigen::Dynamic,Eigen::Dynamic>& Sigma) {

178  return multi\_student\_t\_log<false>(y,nu,mu,Sigma);

179  }

180

181

182  template <class RNG>

183  inline Eigen::VectorXd

184  multi\_student\_t\_rng(const double nu,

185  const Eigen::Matrix<double,Eigen::Dynamic,1>& mu,

186  const Eigen::Matrix<double,Eigen::Dynamic,Eigen::Dynamic>& s,

187  RNG& rng) {

188

189  static const char\* function = "stan::prob::multi\_student\_t\_rng(%1%)";

190

191  using stan::math::check\_finite;

192  using stan::math::check\_not\_nan;

193  using stan::math::check\_symmetric;

194  using stan::math::check\_positive;

195

196  check\_finite(function, mu, "Location parameter", (double\*)0);

197  check\_symmetric(function, s, "Scale parameter", (double\*)0);

198  check\_not\_nan(function, nu,

199  "Degrees of freedom parameter", (double\*)0);

200  check\_positive(function, nu,

201  "Degrees of freedom parameter", (double\*)0);

202

203  Eigen::VectorXd z(s.cols());

204  z.setZero();

205

206  double w = stan::prob::inv\_gamma\_rng(nu / 2, nu / 2, rng);

207  return mu + std::sqrt(w) \* stan::prob::multi\_normal\_rng(z, s, rng);

208  }

209  }

210 }

211 #endif

stan::max\_size\_mvt

size\_t max\_size\_mvt(const T1 &x1, const T2 &x2)

**Definition:** traits.hpp:575

traits.hpp

stan::prob::multi\_student\_t\_log

boost::math::tools::promote\_args< typename scalar\_type< T\_y >::type, T\_dof, typename scalar\_type< T\_loc >::type, T\_scale >::type multi\_student\_t\_log(const T\_y &y, const T\_dof &nu, const T\_loc &mu, const Eigen::Matrix< T\_scale, Eigen::Dynamic, Eigen::Dynamic > &Sigma)

Return the log of the multivariate Student t distribution at the specified arguments.

**Definition:** multi\_student\_t.hpp:35

stan::math::check\_size\_match

bool check\_size\_match(const char \*function, T\_size1 i, const char \*name\_i, T\_size2 j, const char \*name\_j, T\_result \*result)

**Definition:** check\_size\_match.hpp:13

error\_handling.hpp

stan::math::check\_finite

bool check\_finite(const char \*function, const T\_y &y, const char \*name, T\_result \*result)

Checks if the variable y is finite.

**Definition:** check\_finite.hpp:52

stan::scalar\_type::type

scalar\_type\_helper< is\_vector< T >::value, T >::type type

**Definition:** traits.hpp:139

matrix\_error\_handling.hpp

constants.hpp

stan::agrad::lgamma

fvar< T > lgamma(const fvar< T > &x)

**Definition:** lgamma.hpp:15

stan::math::check\_symmetric

bool check\_symmetric(const char \*function, const Eigen::Matrix< T\_y, Eigen::Dynamic, Eigen::Dynamic > &y, const char \*name, T\_result \*result)

Return true if the specified matrix is symmetric.

**Definition:** check\_symmetric.hpp:31

stan::math::check\_ldlt\_factor

bool check\_ldlt\_factor(const char \*function, stan::math::LDLT\_factor< T, R, C > &A, const char \*name, T\_result \*result)

Return true if the underlying matrix is positive definite.

**Definition:** check\_ldlt\_factor.hpp:24

check\_ldlt\_factor.hpp

boost::math::isinf

bool isinf(const stan::agrad::var &v)

Checks if the given number is infinite.

**Definition:** boost\_fpclassify.hpp:54

stan::prob::include\_summand

Template metaprogram to calculate whether a summand needs to be included in a proportional (log) prob...

**Definition:** traits.hpp:35

stan::math::subtract

Eigen::Matrix< typename boost::math::tools::promote\_args< T1, T2 >::type, R, C > subtract(const Eigen::Matrix< T1, R, C > &m1, const Eigen::Matrix< T2, R, C > &m2)

Return the result of subtracting the second specified matrix from the first specified matrix...

**Definition:** subtract.hpp:27

stan::math::LDLT\_factor

**Definition:** LDLT\_factor.hpp:16

stan::agrad::sqrt

fvar< T > sqrt(const fvar< T > &x)

**Definition:** sqrt.hpp:15

stan::prob::inv\_gamma\_rng

double inv\_gamma\_rng(const double alpha, const double beta, RNG &rng)

**Definition:** inv\_gamma.hpp:508

stan::length\_mvt

size\_t length\_mvt(const T &)

**Definition:** traits.hpp:561

stan::math::check\_positive

bool check\_positive(const char \*function, const T\_y &y, const char \*name, T\_result \*result)

**Definition:** check\_positive.hpp:54

dot\_product.hpp

stan::prob::multi\_student\_t\_rng

Eigen::VectorXd multi\_student\_t\_rng(const double nu, const Eigen::Matrix< double, Eigen::Dynamic, 1 > &mu, const Eigen::Matrix< double, Eigen::Dynamic, Eigen::Dynamic > &s, RNG &rng)

**Definition:** multi\_student\_t.hpp:184

stan::prob::multi\_normal\_rng

Eigen::VectorXd multi\_normal\_rng(const Eigen::Matrix< double, Eigen::Dynamic, 1 > &mu, const Eigen::Matrix< double, Eigen::Dynamic, Eigen::Dynamic > &S, RNG &rng)

**Definition:** multi\_normal.hpp:139

stan::math::check\_not\_nan

bool check\_not\_nan(const char \*function, const T\_y &y, const char \*name, T\_result \*result)

Checks if the variable y is nan.

**Definition:** check\_not\_nan.hpp:56

stan::agrad::log\_determinant\_ldlt

var log\_determinant\_ldlt(stan::math::LDLT\_factor< var, R, C > &A)

**Definition:** log\_determinant\_ldlt.hpp:48

size\_

size\_t size\_

**Definition:** dot\_self.hpp:18

stan::VectorViewMvt

**Definition:** traits.hpp:514

stan::prob::multi\_normal\_log

boost::math::tools::promote\_args< typename scalar\_type< T\_y >::type, typename scalar\_type< T\_loc >::type, T\_covar >::type multi\_normal\_log(const T\_y &y, const T\_loc &mu, const Eigen::Matrix< T\_covar, Eigen::Dynamic, Eigen::Dynamic > &Sigma)

**Definition:** multi\_normal.hpp:27

stan::agrad::trace\_inv\_quad\_form\_ldlt

boost::enable\_if\_c< boost::is\_same< T2, var >::value||boost::is\_same< T3, var >::value, var >::type trace\_inv\_quad\_form\_ldlt(const stan::math::LDLT\_factor< T2, R2, C2 > &A, const Eigen::Matrix< T3, R3, C3 > &B)

Compute the trace of an inverse quadratic form.

**Definition:** trace\_inv\_quad\_form\_ldlt.hpp:162

stan::math::log\_determinant\_ldlt

T log\_determinant\_ldlt(stan::math::LDLT\_factor< T, R, C > &A)

**Definition:** log\_determinant\_ldlt.hpp:12

inv\_gamma.hpp

multi\_normal.hpp

stan::math::multiply

boost::enable\_if\_c< boost::is\_arithmetic< T >::value, Eigen::Matrix< double, R, C > >::type multiply(const Eigen::Matrix< double, R, C > &m, T c)

Return specified matrix multiplied by specified scalar.

**Definition:** multiply.hpp:25

subtract.hpp

stan::agrad::log

fvar< T > log(const fvar< T > &x)

**Definition:** log.hpp:15

multiply.hpp

stan::math::dot\_product

double dot\_product(const Eigen::Matrix< double, R1, C1 > &v1, const Eigen::Matrix< double, R2, C2 > &v2)

Returns the dot product of the specified vectors.

**Definition:** dot\_product.hpp:22

matrix.hpp


---

[ Stan Home Page ]

*© 2011–2014,
Stan Development Team.*
